# Measuring Professional Values Among Jordanian and Palestinian Undergraduate Nursing Students: A Comparative Study

**DOI:** 10.1097/jnr.0000000000000580

**Published:** 2023-10-19

**Authors:** Nasser I. ABU-EL-NOOR, Mysoon K. ABU-EL-NOOR, Rabia S. ALLARI

**Affiliations:** 1 PhD, RN, Professor, Faculty of Nursing, Islamic University of Gaza, Palestine; 2 PhD, RN, Associate Professor, Faculty of Nursing, Islamic University of Gaza, Palestine; 3 PhD, RN, Associate Professor, Al-Ahliyya Amman University, Alsult, Jordan.

**Keywords:** professional nursing values, NPVS-R, undergraduate nursing students, Jordan, Palestine

## Abstract

**Background:**

Professional values provide a road map for guiding the behaviors of nursing students during practice and are considered standards for acceptable actions during the provision of nursing care. Nursing educators play a vital role in helping their students embrace professional values in their future career.

**Purpose:**

This study was designed to assess and compare professional values among Jordanian and Palestinian undergraduate nursing students.

**Methods:**

In this descriptive, cross-sectional study, 182 Jordanian and 353 Palestinian nursing students completed the Nurses Professional Values Scale-Revised (NPVS-R), which covers five domains (caring, trust, justice, activism, and professionalism).

**Results:**

The mean age of the participants was 22.5 years, and most (56.6%) were female. The mean total score for the NPVS-R was 3.85, with the “justice” dimension receiving the highest mean score (4.07) and the “activism” dimension receiving the lowest mean score (3.63). The differences in mean NPVS-R total and dimension scores between the Jordanian and Palestinian students were not statistically significant.

**Conclusions:**

The results of this study support that Jordanian and Palestinian undergraduate nursing students have an acceptable level of professional values, with the NPVS-R justice domain scoring relatively high and the NPVS-R activism domain scoring relatively low. The authors hope that the results of this study encourage nursing educators to continue improving professional values among their students, especially with regard to the relatively low-rated dimensions.

## Introduction

The essence of the nursing profession is providing care to the well and sick alike. According to the definition of nursing, “Nursing encompasses autonomous and collaborative care of individuals of all ages, families, groups, and communities, sick or well and in all settings. Nursing includes the promotion of health, prevention of illness, and the care of ill, disabled, and dying people. Advocacy, promotion of a safe environment, research, participation in shaping health policy, and in patient and health systems management, and education are also key nursing roles” (International Council of Nurses, 2002). To perform their tasks well, nurses should abide by the professional values of their profession. Professional values encompass the beliefs, attitudes, and priorities for nurses that serve as a motivation and guide them to interact with their clients, colleagues, and other professionals (Piccone, 2006), and they echo the standards expected when taking specific actions (Schank & Weis, 2001; Weis & Schank, 2009) performed by professional groups such as nursing professionals and individuals. According to Weis and Schank (2009), the way nurses practice their profession with the values of caring, activism, trust, professionalism, and justice has a positive impact on nurse–patient interactions and relationships, patients' safety, and patients' outcomes.

Caring is the cornerstone of nursing. Nurses provide care to the well and the sick alike to the young and the aged at the individual, family, and community levels. As activists, nurses have the role of advocating for patients' rights and having a safe environment. They can participate in shaping health policy to promote justice and overcome health disparities (International Council of Nurses, 2002). Nurses need to build a trusting relationship with their patients. This helps them better understand their needs, therefore ensuring their cooperation with treatment. This in turn reduces patients' stress and improves their outcomes (Habeeb, 2022). The value of professionalism in nursing includes the standard by which nurses measure their performance and reputation, which reflects in nurses' ability to care for their patients successfully, their attitude toward their clients and colleagues, and their ability to work in a team for the good of their clients. It ensures an understanding of the importance of their jobs and to perform their duties in the best possible ways (Habeeb, 2022). Finally, the value of justice is providing needed care to all clients in an equal manner regardless of their age, gender, level of education, race, ethnicity, or religion.

Moreover, these standards are utilized to assess the integrity of an individual or organization, whereas values give a foundation for evaluating professional views and attitudes (Weis & Schank, 1997). Nursing is a profession based on ethical principles and values, and nursing performance is determined by these principles (Poorchangizi et al., 2019). Therefore, values are essential to achieving the goals of nursing practice, which include building a good relationship with clients and colleagues from the healthcare team.

Professional values have long been recognized as essential to practicing as a professional nurse and should be included in any undergraduate nursing program (Nelwati et al., 2019). Although terminology may differ, nurses from different countries follow a shared set of professional values and a comparable set of essential beliefs and attitudes. Respect for client rights including human dignity, beneficence and nonmaleficence, privacy, accountability, and responsibility are just some of these professional values (Weis & Schank, 2009).

Professional value is also central to ethical codes in most nations, including the nursing codes of ethics in Jordan and Palestine, which outline the profession's primary goals, duties, values, and obligations. These codes form and define nurses' commitments to patients and the general public, define the practices of the nursing profession, and clarify the quality inherent to professional nursing care (Weis & Schank, 2009).

Nursing students are the profession's future leaders. Thus, it is critical that they understand and embrace professional principles during their education, which will help them in their future career. The installation and internalization of professional values are critical for the advancement of nursing (Weis & Schank, 2009) and to increasing the capacity of nurses and nursing students for autonomous ethical decision making (Borhani et al., 2010). The role of nursing educator is very important in helping students embrace and internalize professional values, which will guide their behavior and help them conform to related standards in their future practice.

Many studies discussed the importance of professional values from nursing students' perspective. Poorchangizi et al.'s (2019) study aimed to investigate the significance of professional values from the perspective of nursing students. The Nurses Professional Values Scale-Revised (NPVS-R) was used to collect the data from 100 nursing students selected randomly. The results indicated that the students' professional values were highly important, with a mean score of 101.7. “Maintaining the confidentiality of patients” and “safeguarding patients' right to privacy” were the two values that the students felt were most crucial. “Participating in public policy decisions affecting the distribution of resources” and “participating in peer review” were values that the students ranked low in significance (Poorchangizi et al., 2019). Another study conducted by Donmez and Ozsoy (2016) to determine Turkish nursing students' professional values and investigate the connections between each of their traits using the NPVS-R. Four hundred sixteen Turkish nursing students participated in this study. The NPVS-R's overall mean score was determined to be 99.45. The average score was 3.94 for the caring subdimension, 3.84 for the professionalism subdimension, 3.78 for the trust subdimension, and 3.61 for the activist subdimension. The students scored best on the caring subdimension and worst on the activist subdimension.

Numerous studies conducted in a variety of countries have reported on nursing student perceptions of professional values and the correlations between these perceptions and demographic characteristics (Arries, 2020; Bleda et al., 2020; Jahromi et al., 2020). The findings of some of these studies indicate perceptions in the “caring” dimension to be the most highly rated, whereas those in the “activism” dimension are the lowest.

In the Middle East, nursing institutions regularly participate in setting the standards for professional education and practice. In Jordan, core competencies and national standards are a policy document created for all nurses practicing in the country that promotes, guides, and directs the professional nursing practice. This document gives the nursing profession a framework for standardizing general practice and core competencies (Jordanian Nursing Council, 2016). Nursing schools must incorporate these standards into nursing education to train up skilled nurses who possess the necessary core competencies to provide safe, professionally delivered care (Jordanian Nursing Council, 2016).

Although many studies have been published on the professional values of nursing students worldwide, few have been focused on the Arab world. In recent years, only two related studies in Jordan (Allari et al., 2022; Subih et al., 2021) and one in Palestine (Abu-El-Noor et al., 2021) have assessed this issue. Jordan and Palestine share many aspects in common including religion, language, and culture. Therefore, assessing and comparing professional values among Jordanian and Palestinian undergraduate nursing students may add to the related scholarly knowledge.

## Methods

### Design, Setting, Sampling, and Data Collection

A descriptive, cross-sectional design was used in this study. Nursing students enrolled at Jordanian and Palestinian schools of nursing, including bachelor's degree-track students and those who were already holding an associate degree, were targeted as participants. Data were collected between November 1 and December 15, 2021, from one university in each country. Using a convenience sample, 535 students (182 from Jordan and 353 from Palestine) completed the questionnaire. Because of the COVID-19 pandemic, data were collected electronically.

### Instrument

This study relied on the self-administered Nurses Professional Values Scale, which was created by Schank and Weis to assess nursing professional values. The scale was amended by the same authors 9 years later and is now known as the NPVS-R (Weis & Schank, 2009). The NPVS-R was subsequently translated into Arabic by Allari (2016) and consists of 26 items in the five subdomains of caring, activism, trust, professionalism, and justice. Each statement is measured on a 5-point Likert scale ranging from 1 (*not important*) to 5 (*most important*). The Arabic version was found to be valid and reliable (Allari, 2016). In this study, the internal consistency reliability of the NPVS-R was high, with a Cronbach's alpha of .962. The Cronbach's alpha results for the five subdomains were also high, which were as follows: “caring,” .912; “activism,” .939; “trust,” .813; “professionalism,” .804; and “justice,” .800.

### Data Analysis

Data analysis was performed on IBM SPSS Statistics Version 22 (IBM Inc., Armonk, NY, USA). After confirming the accuracy of data entry and data cleaning, the data were analyzed using descriptive statistics (mean, standard deviation, frequency, and percentage). The impact of the study variables on professional values was investigated using Pearson's correlation coefficient, *t* test, and analysis of variance. Differences were considered to be statistically significant at a *p* value of .05.

### Ethical Considerations

The entire study proposal was submitted to and approved by the Research Ethics Committee at the Islamic University of Gaza (Approval No. 35-2020) and the Research Ethics Committee at Al-Ahliyya Amman University, Jordan (Approval No. 2020/2 ف). The aim of the study was presented on the front page of the questionnaire, and participants were assured that their participation was fully voluntary and that they had the freedom to refuse to participate. In addition, the participants were informed that refusal to participate would not result in any penalty or affect their grades. All participant data were obtained anonymously, and confidentiality and anonymity were preserved in the final report.

## Results

### Characteristics of Participants

Five hundred thirty-five undergraduate nursing students from Jordan (*n* = 182, 34%) and Palestine (*n* = 353, 66%) completed the questionnaire. Sociodemographic characteristics and the *p* value of significance between the two countries are presented in Table 1. Participant ages ranged from 18 to 43 years (mean: 21.5 ± 3.1 years). Although most of the participants were women (*n* = 303, 56.6%), the subset of Jordanian participants included more men than women. Most of the participants were enrolled as regular students (*n* = 457, 85.4%), and more than two fifths were in their third year of studies (*n* = 222, 41.5%). The accumulative grade average of participants ranged between 50.2 and 100, with a mean of 78.4 (*SD* = 9.4). Differences in gender, age, and grade average between the participants from the two countries were statistically significant, whereas differences in type of enrollment and year of study were not.

**Table 1.** Participant Characteristics

Variable	All (*N* = 535)		Jordan (*n* = 182)		Palestine (*n* = 353)		*p*
	*n*	%	*n*	%	*n*	%	
Gender							< .001
Male	232	43.4	120	65.9	112	31.7	
Female	303	56.6	62	34.1	241	68.3	
Type of enrollment							.248
Regular student	457	85.4	151	83.0	306	86.7	
Upgrading student	78	14.6	31	17.0	47	13.3	
Year of study							.331
First year	92	17.2	28	15.4	64	18.1	
Second year	75	14.0	32	17.6	43	12.2	
Third year	222	41.5	71	39.0	151	42.8	
Fourth year	146	27.3	51	28.0	95	26.9	
Age (years; *M* and *SD*)	21.5	3.1	22.1	3.5	21.2	2.8	.001
Grade average (*M* and *SD*)	78.4	9.4	75.8	11.7	79.7	7.7	< .001

### The Nurses Professional Values Scale-Revised and Its Dimensions

The mean total score on the NPVS-R was 3.85, with the total score, mean score, and number of items for the NPVS-R and its dimensions shown in Table 2. The “justice” dimension received the highest mean for Jordanian (3.97), Palestinian (4.12), and all (4.07) participants, whereas the “activism” dimension received the lowest mean for Jordanian (3.69), Palestinian (3.61), and all (3.63) participants. Differences among the mean values of NPVS-R and its dimensions were not statistically significant.

**Table 2.** Descriptive Statistics for NPVS-R and Its Domains

Dimension	No. of Items	Total Score	*SD*	Mean ^a^	Rank	*p*
Total score	26					.070
All		100.16	18.83	3.85		
Jordan		99.68	22.50	3.83		
Palestine		100.41	16.60	3.86		
Caring	9				2	.193
All		35.70	7.05	3.97		
Jordan		35.10	8.18	3.90		
Palestine		36.01	6.37	4.00		
Activism	5				5	.298
All		18.17	4.09	3.63		
Jordan		18.45	4.73	3.69		
Palestine		18.03	3.71	3.61		
Trust	5				3	.223
All		19.48	3.66	3.90		
Jordan		19.19	4.28	3.84		
Palestine		19.63	3.29	3.93		
Professionalism	4				4	.053
All		14.60	3.30	3.65		
Jordan		15.01	3.81	3.75		
Palestine		14.39	2.98	3.59		
Justice	3				1	.076
All		12.21	2.48	4.07		
Jordan		11.93	2.81	3.97		
Palestine		12.36	2.28	4.12		

*Note.* NPVS-R = Nurses Professional Values Scale-Revised.

^a^ Total score/number of items for each dimension.

### Caring Dimension

The highest item score in the “caring” dimension was for “maintain confidentiality of patient” (mean = 4.31 ± 0.94), followed by “safeguard patient's right to privacy” (mean = 4.30 ± 0.90). The two lowest item scores in this dimension were for “confront practitioners with questionable or inappropriate practice” and “protect rights of participants in research,” respective with means of 3.69 ±1.08) and 3.65 (±1.07). Only two of the items in the “caring” dimension exhibited significant differences between Jordanian and Palestinian participants (Table 3).

**Table 3.** Mean Scores for Items in the Caring, Activism, Trust, Professionalism, and Justice Dimensions

Domain/Statement	All		Jordan		Palestine		*p*
	Mean	*SD*	Mean	*SD*	Mean	*SD*	
Caring dimension							
Maintain confidentiality of patient	4.31	0.94	4.13	1.06	4.41	0.85	.870
Safeguard patient's right to privacy	4.30	0.90	4.11	1.04	4.39	0.80	.001
Protect moral and legal rights of patients	4.19	0.98	4.03	1.08	4.28	0.91	.009
Practice guided by principles of fidelity and respect for person	4.10	0.94	4.03	1.05	4.14	0.89	.175
Act as a patient advocate	3.87	1.07	3.78	1.14	3.92	1.03	.162
Provide care without prejudice to patients of varying lifestyles	3.84	1.01	3.81	1.10	3.86	0.97	.599
Refuse to participate in care if in ethical opposition to own professional values	3.73	1.18	3.68	1.13	3.76	1.21	.486
Confront practitioners with questionable or inappropriate practice	3.69	1.08	3.80	1.19	3.64	1.02	.112
Protect rights of participants in research	3.65	1.07	3.73	1.13	3.61	1.04	.225
Activism dimension							
Advance the profession through active involvement in health-related activities	4.03	0.99	3.96	1.06	4.06	0.97	.269
Recognize role of professional nursing associations in shaping healthcare policy	3.76	0.98	3.76	1.07	3.76	0.94	.990
Participate in activities of professional nursing associations	3.67	1.09	3.68	1.20	3.66	1.03	.902
Participate in nursing research and/or implement research findings appropriate to practice	3.63	1.08	3.66	1.10	3.62	1.01	.659
Participate in public policy decisions affecting distribution of resources	3.08	1.12	3.39	1.22	2.92	1.03	< .001
Trust dimension							
Maintain competency in area of practice	4.08	0.98	3.89	1.10	4.18	0.90	.002
Seek additional education to update knowledge and skills	3.99	0.97	3.94	1.07	4.02	0.92	.374
Accept responsibility and accountability for own practice	3.96	0.97	3.93	1.05	3.98	0.93	.049
Request consultation/collaboration when unable to meet patient needs	3.93	0.94	3.85	1.06	3.96	0.87	.224
Engage in ongoing self-evaluation	3.51	0.97	3.58	1.09	3.48	0.90	.310
Professionalism dimension							
Initiate actions to improve environments of practice	4.04	0.98	3.91	1.09	4.11	0.91	.028
Promote and maintain standards where planned learning activities for students take place	3.77	1.01	3.81	1.15	3.75	0.92	.543
Establish standards as a guide for practice	3.71	1.04	3.80	1.14	3.66	0.99	.150
Participate in peer review	3.08	1.12	3.51	1.15	2.87	1.05	< .001
Justice dimension							
Protect health and safety of the public	4.31	0.93	4.23	1.05	4.36	0.85	.137
Promote equitable access to nursing and healthcare	4.09	0.99	3.95	1.11	4.17	0.91	.021
Assume responsibility for meeting health needs of the culturally diverse population	3.81	1.02	3.76	1.10	3.83	0.97	.457

### Activism Dimension

The highest scoring item for both Jordanian and Palestinian nursing students in the “activism” dimension was “advance the profession through active involvement in health-related activities” (mean = 4.03 ± 0.999), whereas the item “participate in public policy decisions affecting distribution of resources” received the lowest score (mean = 3.08 ± 1.12). The only statistically significant difference between the Jordanian and Palestinian participants (*p* < .001) in this dimension was the mean score for the item “participate in public policy decisions affecting distribution of resources” (Table 3).

### Trust Dimension

The rank and means for Jordanian, Palestinian, and all participants for the items in the “trust” dimension are presented in Table 3. The highest and lowest mean item scores in this dimension were for “maintain competency in area of practice” (mean = 4.08 ± 0.98) and “engage in ongoing self-evaluation” (mean = 3.51 ± 0.91). No differences in the mean item scores between Jordanian and Palestinian undergraduate nursing students were statistically significant with the exception of the item “maintain competency in area of practice” (*p* = .002)

### Professionalism Dimension

The mean scores for items in the “professionalism” dimension, shown in Table 3, ranged between 4.04 (*SD* = 0.98) for “initiate actions to improve environments of practice” and 3.08 (*SD* = 1.12) for “participate in peer review.” No differences in the mean item scores between Jordanian and Palestinian undergraduate nursing students were statistically significant with the exception of the item “participate in peer review” (*p* < .001).

### Justice Dimension

Finally, the highest scored item in the “justice” dimension for both Palestinian and Jordanian participants was “protect health and safety of the public” (mean = 4.31 ± 0.93). Moreover, the statement “assume responsibility for meeting health needs of the culturally diverse population” had the lowest mean (mean = 3.81 ± 1.02). No differences in the mean item scores between Jordanian and Palestinian undergraduate nursing students were statistically significant (Table 3).

### Influencing Factors on the Nurses Professional Values Scale-Revised and Its Subdomains

The *t* test results revealed that the differences in the means of NPVS-R and its subdomains pertaining to country and type of students' enrolment (regular vs. upgrading) were not statistically significant. In addition, no statistically significant differences in the means of NPVS-R and four of its subdomains were found in relation to students' gender. On the other hand, a statistically significant (*p* = .036) difference in the mean scores for the “trust” dimension between male (mean = 3.82 ± 0.75) and female (mean = 3.95 ± 0.71) participants was detected. The Pearson correlation coefficient test revealed no statistically significant correlations among participant ages or between their accumulative average total scores for the NPVS-R and its subdomains.

The one-way analysis of variance test revealed statistically significant differences among students by year of study in terms of the mean scores for the entire NPVS-R and for the caring, trust, and professionalism subdomains (Table 4). Post hoc results showed that differences in the total score (*p* = .023) and the “caring” (*p* = .038), “trust” (*p* = .006), and “professionalism” (*p* = .008) subdomains were all attributable to differences in the means of first-year and fourth-year students. Differences in mean scores in all of these subdomains favored the fourth-year students (Table 4).

**Table 4.** Descriptive Statistics for NPVS-R and Its Subdomains, by Year of Study

Dimension/Year of Study	Mean	*SD*	*p*
Total score			.021
First	3.65	0.75	
Second	3.87	0.82	
Third	3.87	0.67	
Fourth	3.95	0.72	
Caring			.019
First	3.74	0.84	
Second	4.03	0.88	
Third	3.99	0.74	
Fourth	4.04	0.75	
Activism			.218
First	3.50	0.77	
Second	3.69	0.89	
Third	3.61	0.80	
Fourth	3.72	0.83	
Trust			.005
First	3.68	0.75	
Second	3.86	0.80	
Third	3.91	0.67	
Fourth	4.02	0.72	
Professionalism			.007
First	3.42	0.84	
Second	3.61	0.92	
Third	3.67	0.76	
Fourth	3.79	0.83	
Justice			.144
First	3.92	0.86	
Second	4.00	0.95	
Third	4.09	0.77	
Fourth	4.16	0.80	

*Note*. NPVS-R = Nurses Professional Values Scale-Revised.

## Discussion

The aim of this study was to assess and compare professional values among Jordanian and Palestinian undergraduate nursing students. In general, the results revealed relatively high scores for NPVS-R and its dimensions. For the entire participant sample, the “justice” dimension received the highest mean score and the “activism” dimension received the lowest mean score. This finding is similar to those of Jahromi et al. (2020) and Abu-El-Noor et al. (2021). Differences between Jordanian and Palestinian students in terms of mean scores for the NPVS-R and its respective dimensions were not statistically significant. This may be attributable to the fact that Jordanians and Palestinians share a common religion, language, culture, and moral value system.

Interestingly, the number of Jordanian male participants was greater than that of Jordanian female participants in this study. Over the last two decades, nursing in Jordan has transformed from a female-dominated profession to one in which men outnumber women (Ahmad & Alasad, 2007). AbuGharbieh and Suliman (1992) noted that Jordanian men recognize the worth of the nursing profession and try to enter this profession as a livelihood. Recently, according to Ibrahim et al. (2015), undergraduate male nursing students in Jordan generally hold positive perceptions regarding the image of the nursing profession in the four aspects of the profession's description, society's view, professional benefits, and self-satisfaction. Thus, these may represent major factors currently encouraging male students to enter nursing programs.

The relatively high scores given by the participants for professional values is consistent with the findings of earlier studies conducted in other countries (Arries, 2020; Bijani et al., 2019; Bleda et al., 2020).

The relatively high total NPVS-R scores in this study may be attributable to the nursing programs in both countries providing undergraduate nursing students with the important norms essential to molding and preparing students for future practice as well as establishing and sustaining professional values (Allari et al., 2017). As noted in the comprehensive literature review of Sibandze and Scafide (2018), nurses with a bachelor's degree in nursing are more aware and better able to apply professional values in their practice than nurses with lower levels of education.

The domains receiving the highest scores from all participants were “justice” and “caring.” Justice is considered the “foundation for all professional interactions across all care contexts” (Weis & Schank, 2009, p. 229), reflecting the responsibility of nurses to give equal care to all patients regardless of differences and diversity (Poorchangizi et al., 2019). Moreover, justice is considered one of the most important fundamentals in Islam, which is embraced by the great majority of people in both countries. Islam encourages treating all people equally regardless of race, age, gender, religion, or financial status.

The second most significant domain identified in this study was “caring.” This domain has also been identified in many other studies as either the first or second most significant domain (Allari, 2020; Donmez & Ozsoy, 2016; Sharif et al., 2018). Caring involves “having a concern or attention for that which impacts (another's) wellbeing,” which is an important aspect of nursing practice (Weis & Schank, 2009, p. 229). Furthermore, the American Nurses Association Code of Ethics considers characteristics pertaining to caring to represent the most essential principles and obligations of nurses (American Nurses Association, 2001). According to Begum and Slavin (2012), nurturing a caring attitude among nursing students is critical, as university is the first location where students learn about the profession's most fundamental values and essence. Furthermore, the high scores given by participants on items connected to the “caring” component may reflect their perceptions of caring as the primary function of nurses and as the spirit of nursing practice (Allari et al., 2017). Another reason for the high ratings for the “caring” categories may be the enhancement effect of nursing programs on students' caring ideals and actions (Manninen, 1998) and the practice of evaluating students in their practice primarily on caring skills. The highest item scores in the “caring” dimension were for “maintain confidentiality of patient” and “safeguard patient’s right of privacy.” This clearly reflects the influence of the Islamic religion on the participants from both countries as well as the requirement in educational programs that students give their full attention to nursing practice core skills and competencies. This result echoes the findings of Abu-El-Noor et al. (2021) and Allari (2020).

The “professionalism” and “activism” dimensions earned the lowest mean scores in this study as well as in other prior studies (Green, 2020; Jasemi et al., 2020; Nelwati et al., 2019). According to Jasemi et al. (2020), “activism” is the lowest scored dimension because it is not tied directly to nursing students' clinical practice. Another reason may be that most items in this domain are not included among students' normal clinical-practice evaluation criteria. Despite its importance, the item “participating in research activities and professional association” was valued more by participants who were already involved in clinical practice and teaching nursing students than by those who were more concerned with gaining skills, passing examinations, and getting higher grades in school. The top-rated item in the “activism” dimension in this study was “advance the profession through active involvement in health-related activities,” which may be an indicator of nursing students' empowerment in both countries and a nursing program emphasis on improving the profession. This finding is congruent with many other studies (Jahromi et al., 2020; Paşalak et al., 2021).

A significant difference was found between the means of the item “participate in public policy decisions affecting distribution of resources” for the two nationalities, with the Jordanian students earning a higher mean score. This may relate to the complicated political situation in Palestine, especially in the Gaza Strip, where resources are scarce, and unemployment and poverty rates are very high, which may divert attention away from related activities, as mentioned by Abu-El-Noor et al. (2021).

With the exception of the statement “maintain competency in area of practice,” none of the differences in the mean scores between the Jordanian and Palestinian subgroups was statistically significant in the “trust” dimension. This may be because Palestinian nursing students experience training situations that drive them to cherish the importance of being competent to save lives, especially in emergency and disaster situations, which are very common in Palestine, especially the Gaza Strip, which is subject to repeated attacks from Israel. On the other hand, competency-based training and education is newly applied in nursing programs in Jordan, and faculty members should explore ways to cultivate the importance of competency in practice as a new concept.

The mean score on only one item in the “professionalism” dimension, “participate in peer review,” was statistically different between the two national subgroups, with the Jordanian subgroup scoring significantly higher than the Palestinians. This may be attributable to the Jordanian government's new focus on quality and accreditation in healthcare facilities and academic institutions, making peer review an important approach for quality improvement in nursing in Jordan (Marquis & Huston, 2009). Therefore, many universities in Jordan, including nursing faculties, have adopted new evaluation strategies, including peer review, in the courses they offer, especially in clinical courses.

The top statement rated by participants from both countries in the “justice” dimension was “protect health and safety of the public.” Patient-related safety activities are potentially the most important indicator of professionalism in nursing practice, and in both Jordan and Palestine, seeking public safety and health protection is a fundamental doctrine of the ethical nursing practice (Abu-El-Noor et al., 2021; Allari et al., 2017).

With regard to the factors found to influence the NPVS-R and its subdimensions, neither country nor type of student enrolment (regular vs. upgrading) was found to have a significant impact. Participants from both countries seemed to be alike in many qualities, which reflects their sharing the same geographical area, culture, beliefs, and religion. On the other hand, although there were no statistically significant differences in the means of NPVS-R and four of its subdomains with regard to gender, the difference in the “trust” dimension was statistically significant, with female participants earning a higher mean score than their male peers. Nursing in many countries is still dominated by women, and in this study, the number of female participants was much higher than that of male participants (56.6% vs. 43.4%). In Arab–Muslim countries, children, especially daughters, are raised to value trust as a protective behavior. The concept of trust is highly valued and prioritized and includes taking responsibility and being accountable for one's own behaviors. Thus, this concept is naturally reflected in the professional values of Arab–Muslim women. This result was similar to that of Paşalak et al. (2021), who reported that women earned higher scores in the domain of “trust,” and contradicts that of Schmidt (2016), who found male students earned a higher mean score for trust than female students.

Nevertheless, each value statement in all NPVS-R dimensions is equally important and should be considered so during the education process. Nursing faculties and educators must attempt to enhance all of the dimensions of professional values, develop appropriate teaching and training strategies, and act as role models by focusing on all aspects of values, especially those items receiving lower scores (Chikeme et al., 2019).

In this study, statistically significant differences in mean scores were found for year of study for the NPVS-R and the subdomains of “caring,” “trust,” and “professionalism.” This is similar to the findings of previous studies (Kantek et al., 2017; Paşalak et al., 2021). The mean scores for fourth-year students were higher than those for first-year students, which is expected in Benner's novice-to-expert model, which applies the stages of clinical competency (Fisher, 2014). The highest differences being in the previous domains (caring, trust, and professionalism) may be attributable to advanced-year students viewing the caring relationship with the patients and being professional as more important than advancing the healthcare system, as mentioned by Allari et al. (2017). This situation may be also attributable to advanced-year students having greater exposure to clinical practice involving a variety of roles and responsibilities and thus exhibiting higher levels of emotional intelligence, which plays a positive role in value appreciation (Bleda et al., 2020). Moreover, first-year students have not directly experienced patient care. Therefore, greater emphasis should be placed on the importance and necessity of cultivating professional values among the nursing students during the first year of their education. Nursing curricula should be reviewed and discussed in all nursing programs in both countries with regard to how to better integrate professional values in all courses, raise awareness, and engage and guide students starting from their first year in the program to develop professional values. Conferences and forums may be organized with faculty members, nursing pioneers, and nurse activists involved in public advocacy and policymaking. In addition, students should be encouraged to participate in the organization and implementation of national nursing events and community activities, peer reviews, self-evaluations, and personal reflections.

### Conclusions and Implications for Practice

In this study, the respective levels of professional values among Jordanian and Palestinian undergraduate nursing students were similar with the exception of several subdimensions. Professional values were considered important by students of all academic years and especially by those in their fourth year. Therefore, to encourage the professional development and transformation of junior students' value system into future professional nurses, reviewing and integrating professional values in nursing curricula and developing appropriate teaching methods to cultivate, improve, and promote professional values among nursing students should be strategic objectives adopted by all nursing programs.

A key nursing education standard/outcome related to professional values in higher education is the understanding of and adherence of students to professional values and ethical behaviors during practice. Nursing programs can accomplish these goals by giving students the opportunity to join organizations that advocate high standards of practice in healthcare and participate in interprofessional service learning projects, by using realistic scenarios to teach students about ethical and legal patient care situations, and by identifying outcomes using reflective writing as a strategy to evaluate students' perceptions of the role of advocacy and identify the influence of their attitudes and values on practice to develop a student honor code and professional values and show professionalism (Accreditation and Quality Assurance Commission, 2014). Moreover, nursing educators must support students by serving as role models, empowering them, and getting them involved in additional activities to strengthen these values.

The results of this study may help nursing program administrators better integrate professional values into their curricula, which will improve student competencies, achieve desired nursing program outcomes, and improve client safety and quality of care. Longitudinal studies may be conducted to follow student progress during their 4-year education to further elucidate the factors that influence the development of professional values in nursing students.
